# Injectable pH Thermo-Responsive Hydrogel Scaffold for Tumoricidal Neural Stem Cell Therapy for Glioblastoma Multiforme

**DOI:** 10.3390/pharmaceutics14102243

**Published:** 2022-10-20

**Authors:** Jasmine L. King, Panita Maturavongsadit, Shawn D. Hingtgen, S. Rahima Benhabbour

**Affiliations:** 1 Division of Pharmacoengineering and Molecular Pharmaceutics, Eshelman School of Pharmacy, University of North Carolina, Chapel Hill, NC 27599, USA; 2 Joint Department of Biomedical Engineering, University of North Carolina and North Carolina State University, Chapel Hill, NC 27599, USA

**Keywords:** neural stem cell, stem cell engineering, stimuli responsive hydrogels, thermo-responsive hydrogels, glioblastoma multiforme

## Abstract

Glioblastoma multiforme (GBM) is the most common malignant brain tumor in adults and despite recent advances in treatment modalities, GBM remains incurable. Injectable hydrogel scaffolds are a versatile delivery system that can improve delivery of drug and cell therapeutics for GBM. In this report, we investigated an injectable nanocellulose/chitosan-based hydrogel scaffold for neural stem cell encapsulation and delivery. Hydrogels were prepared using thermogelling beta-glycerophosphate (BGP) and hydroxyethyl cellulose (HEC), chitosan (CS), and cellulose nanocrystals (CNCs). We evaluated the impact of neural stem cells on hydrogel gelation kinetics, microstructures, and degradation. Furthermore, we investigated the biomaterial effects on cell viability and functionality. We demonstrated that the incorporation of cells at densities of 1, 5 and 10 million does not significantly impact rheological and physical properties CS scaffolds. However, addition of CNCs significantly prolonged hydrogel degradation when cells were seeded at 5 and 10 million per 1 mL hydrogel. In vitro cell studies demonstrated high cell viability, release of TRAIL at therapeutic concentrations, and effective tumor cell killing within 72 h. The ability of these hydrogel scaffolds to support stem cell encapsulation and viability and maintain stem cell functionality makes them an attractive cell delivery system for local treatment of post-surgical cancers.

## 1. Introduction

Glioblastoma multiforme (GBM) is the most aggressive form of brain cancer and 90% of GBM patients die within 12 to 15 months after diagnosis [1]. Approximately, 13,000 patients are newly diagnosed with GBM every year [2]. The current clinical standard of care consists of maximal surgical resection followed by adjuvant chemoradiation [1,3,4]. Despite these efforts, GBM recurrence is inevitable. This is likely due to a subpopulation of cells within the tumor that express characteristics of stemness which ultimately plays a key role in promoting tumor growth and conferring chemo-resistance [5]. Furthermore, given the diffusely infiltrative nature of GBM, small molecule drugs are unable to travel to these distant lesions.

Recently, neural stem cell (NSC) therapy has shown promise as a new approach at targeting GBM foci. Genetically engineered NSCs possess inherent tumor-tropic properties that allows them to migrate toward and deliver therapeutic gene products such as proapoptotic agent tumor necrosis factor-α (TNF-α)-related apoptosis-inducing ligand (TRAIL) to local or distant GBM foci. Preclinical studies have demonstrated the ability of these tumoricidal NSCs to blunt tumor growth and extend median survival in post-surgical GBM xenograft model [6,7,8]. A transdifferentiation process was developed by Hingtgen et al. that allows for human fibroblast to be transformed into human induced neural stem cells [8,9]. This method is achieved by utilizing a single SOX-2 transcription factor strategy. For a more clinically translational approach, human fibroblast has been obtained from cancer patients, reprogrammed into tumor-homing iNSCs, functionally profiled, and assessed for their interpatient variability [10]. Although there are advantages and unique features associated with this stem cell carrier, intracranial administration of these stem cells results in (1) rapid stem cell clearance, (2) poor stem cell retention, and (3) poor stem cell persistence in the post-surgical cavity which remains a major challenge that limits their clinical utility.

Biodegradable polymer implants have long been used to improve delivery of chemotherapeutics in patients with primary and recurrent GBM [11,12]. Among these applications, polymer-based biomaterials have been utilized to improve retention and efficacy of neural stem cells within the post-surgical cavity [13,14,15]. However, some of these approaches have been limited to cell seeding methods which have several concerns that may affect treatment efficacy such as number of cells attached, homogeneity, and inconsistent dosing. Furthermore, modifications to the scaffold properties such as morphology, diameter, porosity, and size did not have a significant clinical impact on efficacy [15]. Given the limitations of these approaches, there have been other strategies aimed at exploring biomaterials that can (1) encapsulate neural stem cells and (2) maintain a physiological environment applicable for neural stem cell viability and retention following implantation [16,17,18]. Preclinical studies utilizing commercially available synthetic extracellular-matrix based materials have shown to increase NSC persistence from 2 weeks to 95 days [18] and extend overall survival in mice following implantation [16].

While these results are promising, data defining the impact of scaffold properties on NSC persistence, release, and overall ability to migrate to distant GBM foci given the degradation profile is limited. Although it is important to utilize scaffolds that consist of polymers that are biocompatible and cytocompatible, there is a significant interest in selecting polymers with properties that (1) can allow for maximum stem cell retention following implantation; (2) have the ability to fine-tune scaffold degradation; and (3) control the release of therapeutic NSCs from the matrix. To address some of these concerns, we have developed an injectable thermo-/pH-responsive cellulose nanocrystal (CNC)-hybridized chitosan (CS)-based hydrogel system as a scaffold for stem cell encapsulation and delivery while maintaining cell viability overtime [19,20]. The thermosensitive nature of CS-based hydrogels allows for the injectable solution to undergo a sol-gel transition into a gel once exposed to physiological conditions.

Biomaterials that can improve the clinical utility of NSC therapy for GBM are in high demand. In this study, we investigated the use of this injectable pH-/thermo-responsive biomaterial to improve NSC delivery. To this end, we explored two hydrogel formulations 1) CS only and 2) CS with the addition of CNCs. To our knowledge, there are no technologies that combine the use of therapeutic NSCs and CS-based hydrogels for brain cancer therapy. The objective of this work aimed to assess the impact of our biomaterials on NSC viability and functionality as well as the impact of NSCs on the rheological and degradation properties of the CS and CS-CNC hydrogel scaffolds. This work will establish the novel use of NSC-laden CS hydrogels for post-surgical GBM and has the potential for broader range of applications including regenerative medicine and other solid organ tumors.

## 2. Materials and Methods

### 2.1. Materials

Chitosan (CS) powder (85% deacetylated, 200–800 cP, 3 wt% in 0.1 M acetic acid), β-glycerophosphate (BGP), and hydroxyethyl cellulose (HEC, MW: 90,000 Da) were purchased from Sigma-Aldrich (St. Louis, MO, USA). LIVE/DEAD^®^ Viability/Cytotoxicity kit containing Calcein AM and Ethidium Homodimer-1 reagents were purchased from ThermoFisher Scientific (Waltham, MA, USA). 1 mL Luer-Lock syringes were purchased from Becton and Dickinson (Macquarie Park, Australia). Luer-Lock connectors were purchased from Baxter (Deerfield, IL, USA).

#### 2.1.1. Cell Lines 

U87-MG tumor cells and C17 mouse neural stem cells (NSCs) were obtained from the American Type Culture Collection (ATCC). U87-MG and C17 cells were cultured in Dulbecco’s Modified Eagle Medium (Gibco) supplemented with 10% fetal bovine serum (FBS; Gibco), 1% penicillin/streptomycin (P/S; Gibco) and 4 mM L-glutamine (referred to as standard cell culture media). The hTERT-immortalized normal human fibroblasts (NHF-1) were obtained from W. Kauffmann (University of North Carolina School of Medicine).

#### 2.1.2. Lentiviral Vectors

The following lentiviral vectors (LVs) were obtained from Duke University Viral Vector Cores (Durham, NC, USA): reverse tetracycline-controlled transactivator (rtTA), doxycycline inducible SOX2 (SOX2), mCherry-Firefly Luciferase (mCh-FLuc), and a green fluorescent protein fused with a secretable TRAIL variant (GFP-sTRAIL).

### 2.2. Methods

#### 2.2.1. Transduction of Cell Lines

C17, U87-MG, and NHF-1 cells were transduced with virus at varying multiplicities of infection and incubated overnight at 37 °C/5%CO_2_ with standard cell culture media containing 1 g/mL polybrene [21]. Twenty-four hours following incubation, the virus containing media was replaced with fresh standard cell culture media; and thereafter, fluorescent signal was monitored.

#### 2.2.2. Generation of iNSCs

Therapeutic iNSCs were generated from the following established protocol [21]: On day 1, 2 × 10^6^ virally infected NHF-1 cells were plated in a T175 tissue culture flask, supplemented with standard cell culture media, and incubated at 37 °C/5%CO_2_ for 24 h. Post 24-h incubation, the standard cell culture media was removed and replaced with neural induction media containing 2 μg/mL doxycycline (referred to as transdifferentiation media). The transdifferentiation media was replenished every other day for 5 days. On day 5, cells were harvested using Accutase Cell Detachment Solution (STEMCELL Technologies, Vancouver, BC, Canada) and filtered through a 100 μm cell strainer.

#### 2.2.3. Preparation of Cellulose Nanocrystal-Hybridized Chitosan-Based Hydrogels

A 3% *w*/*v* chitosan (CS) stock solution was prepared as previously reported by stirring chitosan powder in 0.1 M acetic acid in deionized water at room temperature for 48 h [19]. The 1 M β-glycerophosphate (BGP) stock solution was prepared by dissolving BGP in deionized water. Hydroxyethyl cellulose (HEC) stock solution (25 mg/mL) was prepared by dissolving HEC in DMEM media. The synthesis of the in situ forming, thermogelling hydrogels was performed using a three component, two-step mixing system under aseptic conditions. The three separate components consisted of: (1) CS, (2) BGP, (3) HEC, cellulose nanocrystals (CNCs), with or without stem cells in DMEM media. In brief, the first 2% *w*/*v* CS and BGP gelling component system were combined and homogenously mixed using a Luer Lock cap. The third component system was transferred into the CS system to create a pre-hydrogel mixture, followed by incubation at 37 °C for 30 min to create a hydrogel.

#### 2.2.4. Determination of Hydrogel Gelation Time

Gelation times of CS-based hydrogels were determined using a rotational rheometer, DHR-3 (TA Instruments, New Castle, DE, USA) 24 h after preparation. Rheology measurements were made using an 8 mm diameter parallel plate. The temperature was controlled at 37 °C by Peltier Plate heating system. The samples were placed on to the lower plate surface and the upper plate was lowered to a 0.5 mm gap distance. Optimization of strain amplitude (0.01%, 0.05%, and 0.1%) and angular frequency (10, 15, and 20 rad/s) showed that optimal results within the linear viscoelastic region of the hydrogel were obtained with 0.05% strain amplitude and angular frequency of 20 rad/s. As such, for all rheological measurements, the dynamic time sweep was performed at 0.05% strain with an angular frequency of 20 rad/s.

#### 2.2.5. In Vitro Mass Loss Analysis

The hydrogels were prepared as previously described using 1 mL Luer Lock syringe [19]. Briefly, two hundred microliters (200 μL) of pre-hydrogel mixture was injected into a syringe mold using an 18 G needle (200 μL/mold, *n* = 6) and incubated at 37 °C for 30 min. The hydrogel samples were subsequently transferred from the molds and seeded directly into a 12-well plate and cultured in DMEM media. On days 0, 7, 14, and 30, the hydrogel samples were collected, lyophilized for 24 h using FreeZone 4.5 Liter Freeze Dryer (Labconco, Kansas City, MO, USA), and weighed to determine the mass loss using the following equation: % Mass loss (%) = (W*_i_* − W*_f_*)/W*d* × 100; W*_i_*—initial weight at day 0; W*_f_*—final weight at given time point.

#### 2.2.6. Scanning Electron Microscopy (SEM)

Hydrogels were prepared as previously described with a cell loading density of 10^6^–10^7^ NSCs/mL of hydrogel (200 μL/mold, *n* = 3) and incubated at 37 °C/5%CO_2_ in serum free DMEM media for 24 h. The hydrogel scaffolds were removed, flash frozen with liquid nitrogen, and lyophilized for 24 h. Lyophilized samples were subsequently sliced, mounted onto carbon tape, and sputter coated with 8 nm of gold-palladium alloy. Images were captured using a Zeiss Supra 25 field emission scanning electron microscope with a numerical aperture of 30 µm at an average working distance of 10 mm (Carl Zeiss Microscopy, LLC, Thornwood, NY, USA).

#### 2.2.7. Stem Cell Viability

To determine cell viability, NSCs (1 × 10^6^ cells/mL) were encapsulated in 1 mL of pre-hydrogel mixture and seeded directly into an 8-well chamber glass slide (50 µL/well, *n* = 4). The samples were incubated in standard cell culture media for 30 min at 37 °C to form a hydrogel. On day 1, 7, 14, 30, after seeding, cell viability was assessed using LIVE/DEAD^®^ Viability/Cytotoxicity kit (Invitrogen, Waltham, MA, USA). The fluorescent images were captured at 5× magnification using a laser scanning confocal microscope (Zeiss LSM700, Jena, Germany). 10 z-stacks were collected per sample.

#### 2.2.8. In Vitro TRAIL Release

CS/CS-CNC hydrogels were prepared as previously described with a cell loading density of 10^6^–10^7^ iNSC-s-TRAIL/mL of hydrogel (100 μL/mold, *n* = 7) and incubated at 37 °C/5%CO_2_. Hydrogel samples were prepared in individual molds and subsequently seeded in a 12-well plate and supplemented with standard cell culture media for 24 h. Following incubation, 1 mL aliquot of TRAIL conditioned media was collected. To measure the amount of TRAIL released from the cell-laden hydrogel scaffolds, an in vitro Human TRAIL Enzyme-Linked Immunosorbent Assay (ELISA) was performed following manufacturer’s protocol (Abcam ab46074, Cambridge, UK). The absorbance from each well was quantified using a microplate reader and the concentration of secreted TRAIL (s-TRAIL) in each sample was determined based on an established standard curve.

#### 2.2.9. In Vitro Cell Kill

U87-MG cells expressing mCh-FLuc (1 × 10^4^ cells/well) were seeded in 96-well plate using standard cell culture media. The cells were incubated for 24 h to allow attachment. iNSCs-sTRAIL were generated as previously described and encapsulated in the hydrogel precursor formulation at the following cell loading densities: 1 × 10^6^, 5 × 10^6^, 1 × 10^7^ per mL of hydrogel, respectively. Following encapsulation, the cell-laden hydrogel precursor formulation was seeded into a 1 mL inkjet syringe mold and incubated at 37 °C/5%CO_2_ for 30 min (100 μL/mold; *n* = 6). After gelation, the cell-laden hydrogels were seeded into a 12-well plate containing 1 mL of standard cell culture media. After 24- and 72-h incubation, standard cell culture media containing sTRAIL (sTRAIL conditioned media) was added to each well containing U87-MG cells (100 μL/well; *n* = 6). Tumor cell viability was measured 24 h later by CellTiter-Glo^®^ Cell Viability Luminescent Assay (Promega, Madison, WI, USA).

### 2.3. Statistical Analysis

Statistical analysis was performed using two-way ANOVA multiple comparisons test with GraphPad Prism Software v9.3.1.

## 3. Results

### 3.1. Injectable Chitosan-Based Hydrogels for NSC Encapsulation

**Figure 1:**
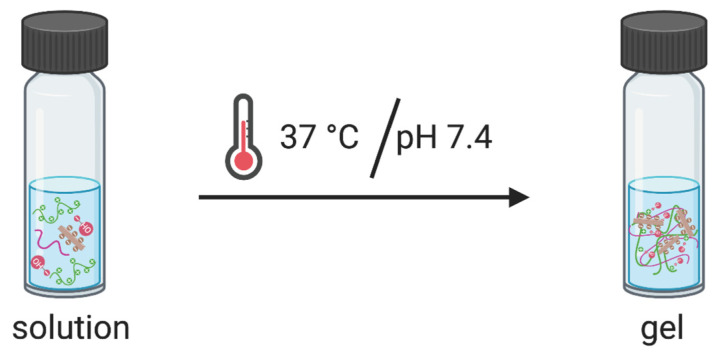
Schematic illustration of sol-gel transition and polymer crosslinking behavior under physiological conditions at 37 °C and pH 7.4.

CNC-hybridized chitosan-based hydrogels that undergo a sol-gel transition process in response to temperature and pH changes were prepared using a previously reported procedure (Figure 1) [19].

**Figure 2:**
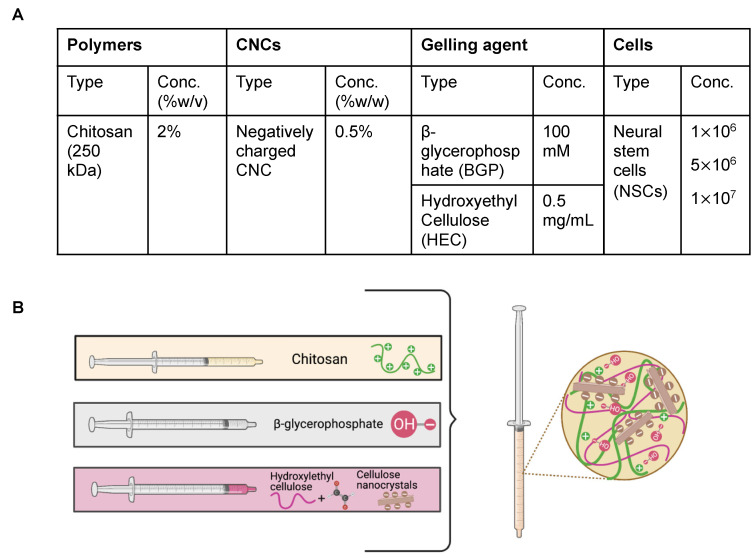
Optimized hydrogel formulations used in this study. (**A**) Table representing components of hydrogel formulations. (**B**) Schematic illustration of polymer interactions within hydrogel matrix.

The optimized formulation composition is illustrated in Figure 2. To achieve appropriate osmolarity (300–400 mOsm/kg) and pH for cell survival (6.5–7.4), 100 mM BGP was used as the primary gelling agent [19,22,23]. HEC surface-treated with glyoxal was incorporated as secondary gelling agent at an optimized concentration of 0.5 mg/mL to promote faster gelling kinetics. We have previously shown that the reactive glyoxal molecule in HEC covalently cross-links to the CS backbone via a Schiff base reaction between the aldehyde functional group in the glyoxal molecule and the deprotonated amine functional group in CS [19]. Based on our previous findings, CNCs were incorporated at 0.5% *w*/*v* as a reinforcing agent and to enhance gelation kinetics and prolong hydrogel degradation, which can as a result lead to sustained release of cells from the hydrogel scaffolds.

### 3.2. In Vitro Characterization of CS-CNC Hydrogel Scaffolds

Given that the sol-gel transition of our prototype hydrogels is facilitated by primary physical crosslinking and secondary chemical crosslinking that occurs under physiological conditions, we set out to investigate the effects of encapsulated cells when seeded at different densities on the physical and chemical properties of the polymeric network of hydrogels. To determine the optimal cell density in hydrogel formulations, we investigated the gelling kinetics, physical properties, and degradation profiles of CS and CS-CNC hydrogels following cell encapsulation at varying cell densities. Initial formulation screening process was done with CS-only hydrogels and consisted of 1 million to 50 million NSCs per mL of hydrogel. Results showed that 1, 5, and 10 million cells per mL of hydrogel resulted in uniform homogenous pre-hydrogel mixtures following encapsulation. Higher cell densities (>15 million/mL) resulted in non-homogenous mixing, increased hydrogel viscosity, and loss of syringeability. Therefore, these cell densities were not further characterized in our CS-CNC scaffolds. Details of the formulation screening process is summarized in Supplementary Figures S1 and S2.

**Figure 3:**
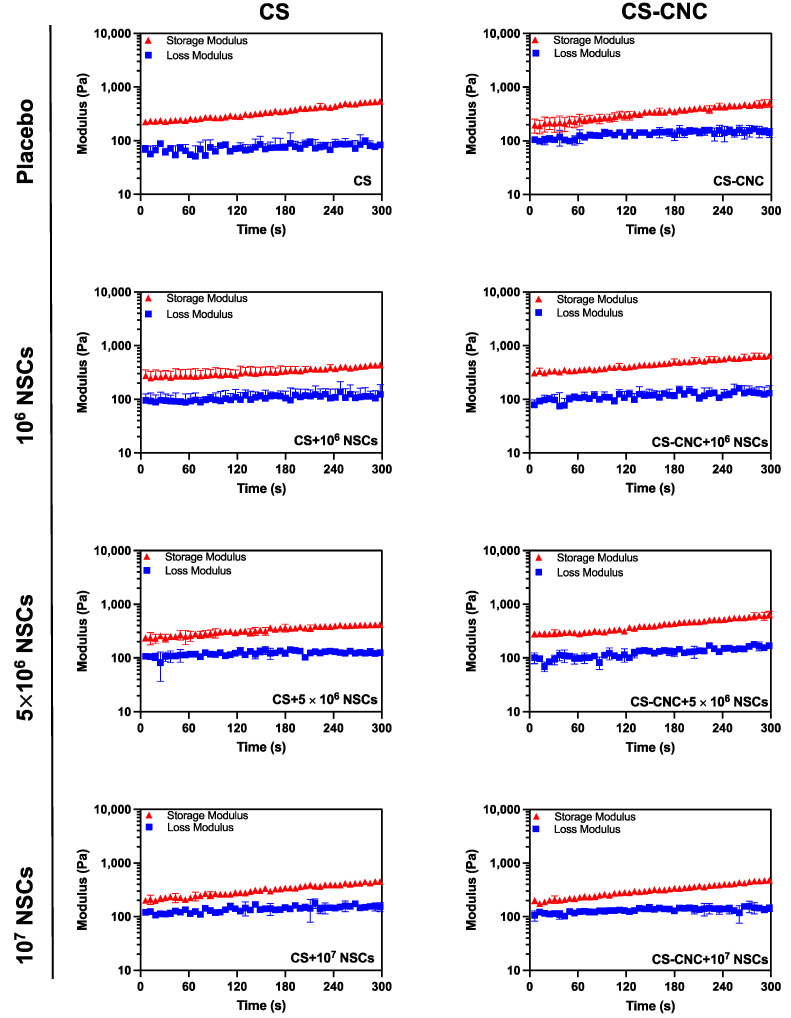
Effect of NSCs on hydrogel gelation kinetics. Gelation time of injectable cell-laden hydrogels determined by time at crossover G’ and G” (*n* = 3).

Rheological analysis to determine the gelation kinetics was conducted on all optimized NSC-laden hydrogel scaffolds. Gelation kinetics are a critical parameter that determines the ability of the hydrogel scaffolds to maintain their shape and retain encapsulated cells at the site of injection (i.e., GBM resection cavity). The gelation kinetics were observed by evaluating the variation in dynamic response at a constant temperature, angular frequency, and strain. This can be further defined as the measurement time at which the storage modulus (G′) and loss modulus (G″) reach a crossover point. The NSC-laden hydrogel scaffolds exhibited instantaneous gelation indicated by a gelling time of less than 7 s (Figure 3). The fast gelation observed in both placebo and stem-cell laden hydrogel scaffolds demonstrated that there was no significant impact on gelling kinetics with the presence of NSCs at their respective cell loading densities.

**Figure 4:**
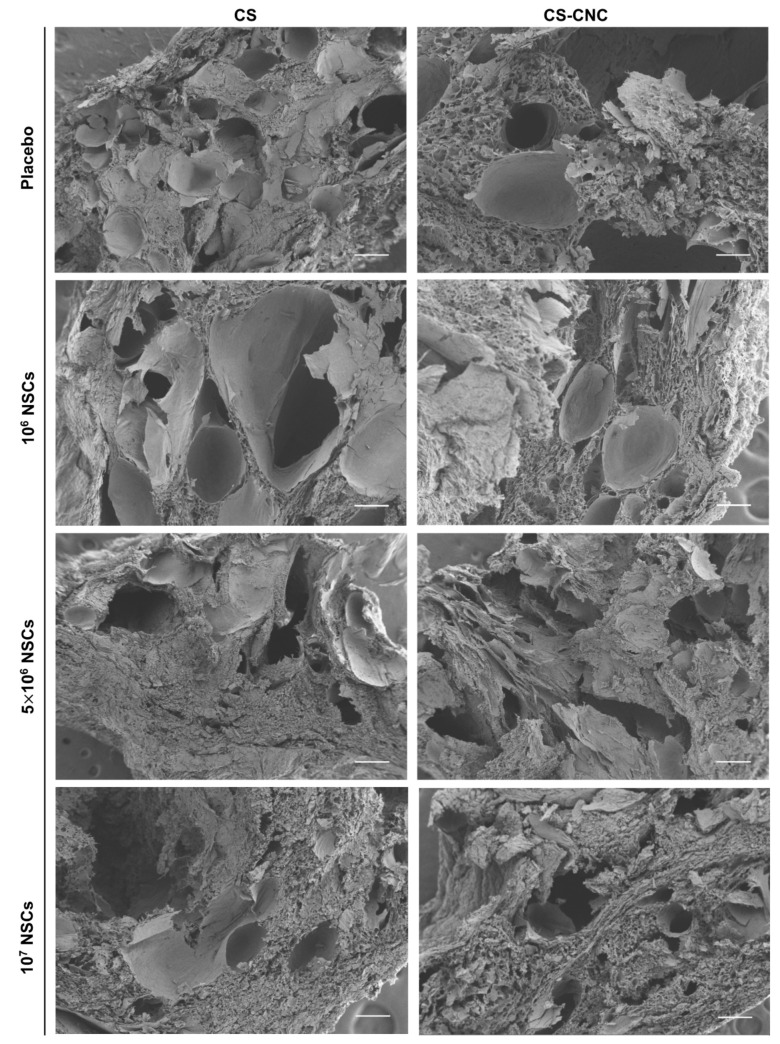
Effect of NSCs on scaffold microstructure using Scanning Electron Microscopy. SEM images of hydrogels post 24-h incubation in DMEM (scale bar, 100 μm).

The microscopic features of hydrogels are important to determine the degree of porosity to allow for diffusion of cells, secretable therapeutic proteins or molecules from cells, and nutrients for cell survival. SEM imaging was performed to observe the microstructure of CS-CNC and NSC-laden CS-CNC scaffolds following gelation. SEM images were taken for hydrogels at 24 h post incubation in standard cell culture media at 37 °C/5%CO_2_, and results showed that hydrogel scaffolds had a highly porous microstructure (Figure 4). Based on the SEM images, there were no noticeable differences in the hydrogel microstructures as a function of cell density and/or addition of CNCs (length: 100–200 nm; zeta potential: −14.7 mV ± 0.11) [19]. Therefore, our results demonstrated that all hydrogel scaffolds should similarly be able to support diffusion functions related to cell survival and delivery.

**Figure 5:**
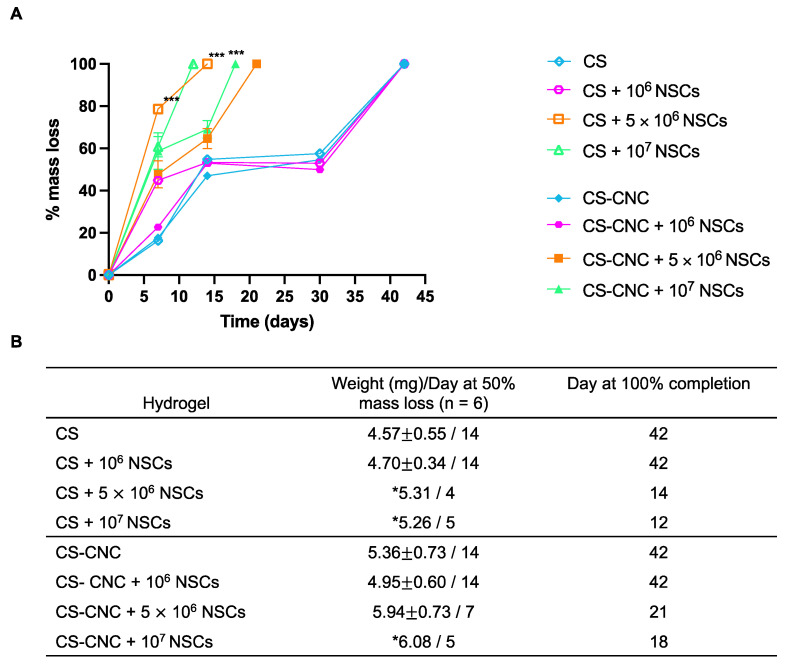
Effect of NSCs on hydrogel degradation over time. (**A**) Percent mass loss of CS-CNC hydrogels with and without NSCs (*n* = 6, 200 µL samples; *** indicates *p* < 0.0001). (**B**) Summary table of mass at 50% degradation, time to 50% mass loss (days), and time to 100% completion (days) (mass represented as mean ± standard deviation (SD); * indicates weight obtained from extrapolated hydrogel mass at 50% mass loss).

The effect of NSCs on the degradation of CS and CS-CNC hydrogel scaffolds was also investigated. Hydrogel degradation plays key roles in retaining therapeutic NSCs at the resection site and governing the release of therapeutic NSCs from the hydrogel matrix. It is known that CS-based hydrogels undergo degradation via enzymatic or hydrolytic mechanisms [24]. Based on our previous studies, we hypothesized that the incorporation of CNC nanomaterial would provide the ability to fine tune the degradation rate of CS-based hydrogels and therefore sustain the release of cells from the hydrogel matrix [19,20]. Cell-free and NSC-laden CS and CS-CNC hydrogel scaffolds were prepared, incubated in standard culture media at 37 °C/5%CO_2_, and assessed for decrease in mass of hydrogel at various time points. As shown in the summary graph, there was no significant difference in degradation when comparing cell-free CS- and CS-CNC hydrogel scaffolds at all time points, respectively (*p* > 0.9999; two-way ANOVA multiple comparisons) (Figure 5). Similarly, no significant difference in degradation profiles was observed with CS and CS-CNC hydrogel scaffolds seeded with 1 million NSCs (*p* > 0.9999; two-way ANOVA multiple comparisons) (Figure 5). However, all NSC-laden hydrogel scaffolds seeded with 5 or 10 million cells exhibited statistically significant differences (*p* < 0.0001, two-way ANOVA multiple comparisons) at varying time points. Specifically, notable differences were observed in CNC-containing NSC-laden hydrogel scaffolds when compared to CS only analogues. As illustrated in Figure 5B, the time to 100% degradation was extended for 5 and 10 million NSC-laden hydrogel scaffolds that contained CNCs in comparison to hydrogel scaffolds without CNCs (21 vs. 14 days for 5 million cells/mL; 18 vs. 12 days for 10 million cells/mL).

### 3.3. In Vitro Cell Studies

**Figure 6:**
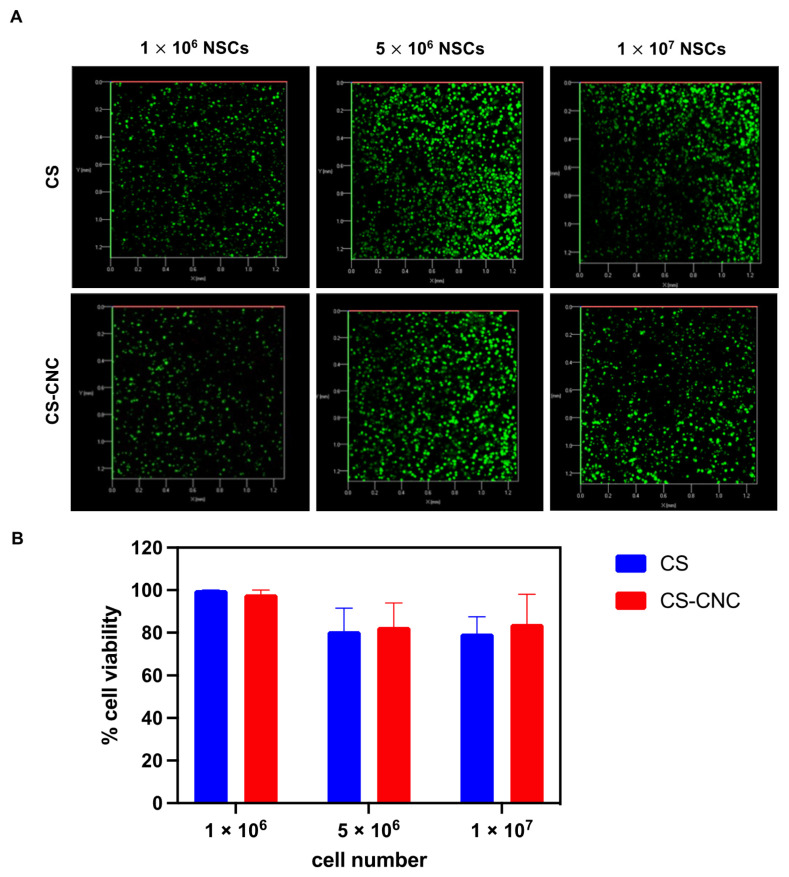
In vitro cell viability within hydrogel matrices. (**A**) Confocal fluorescent images of NSC-hydrogels (10^6^–10^7^ NSCs/mL hydrogel) post 24 h incubation (*n* = 3; 200 μL samples; *x*- and *y*-axis 0.0–1.2 mm). (**B**) Graphical representation of NSC viability.

Cell viability was assessed to determine the cytocompatibility and biocompatibility CS and CS-CNC hydrogels. Previously, we demonstrated the ability to maintain viability of MC3T3-E1 cells in the CS and CS-CNC hydrogel formulations at a cell seeding density of 1 million cells per mL of hydrogel [19]. Here, we report the qualitative evaluation of cell viability by live image analysis using confocal microscopy. NSCs were encapsulated in the CS and CS-CNC hydrogel formulations at the cell densities described above, incubated in standard cell culture media at 37 °C/5%CO_2_, and imaged to determine live vs. dead cells after 24 h incubation. As illustrated in Figure 6, results indicate similar cell viability when comparing CS and CS-CNC hydrogel scaffolds at their respective cell densities, demonstrating that the presence of CNCs did not affect cell viability. The percentage of live and dead cells was imaged and quantified using ImageJ analysis (Figure 6A and Figure S3). Graphical representation of those results is shown in Figure 6B. NSCs seeded at 1 million demonstrated 98–100% cell viability in both CS and CS-CNC hydrogels. However, at seeding densities of 5 and 10 million cells/mL NSCs showed a slight decrease in cell viability, resulting in 80–84% cell viability in both hydrogel formulations. These results demonstrate that both the CS and CS-CNC hydrogel scaffolds are suitable formulations for maintaining cell viability. Further in vivo cell viability testing is warranted to better understand the impact of cell viability over time.

**Figure 7:**
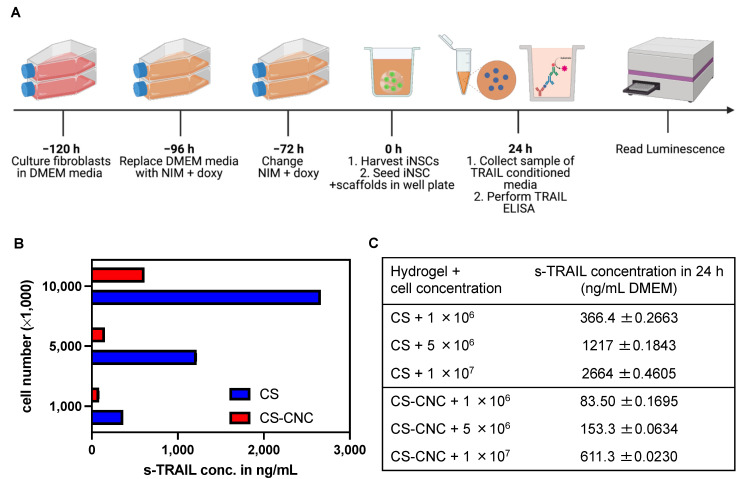
Impact of CS-CNC hydrogels on s-TRAIL release. (**A**) Schematic illustration of in vitro s-TRAIL release experimental design. (**B**) Graphical representation of s-TRAIL concentration released from iNSC-hydrogels post 24-h incubation (*n* = 7; 100 μL samples). (**C**) Summary table of s-TRAIL concentration from cell-laden hydrogel scaffolds.

To determine the effect of CS and CS-CNC hydrogels on the release of therapeutic protein (TRAIL) from the engineered stem cells, we investigated the in vitro release of TRAIL using a sandwich-based ELISA. As previously stated, the porosity of hydrogels plays a key role in mediating the diffusion of nutrients into the matrix as well as the diffusion of cells and/or secreted therapeutic proteins or molecules from cells out of the matrix. To take a step towards human patients, we utilized a more clinically relevant cell line, human iNSCs, to carry out in vitro release studies. Results showed that there were notable differences in TRAIL levels when the iNSC-sTRAIL-laden CS and CS-CNC hydrogels were compared (Figure 7A). Increase in cell density resulted in an increase in the amount of TRAIL released from both CS and CS-CNC hydrogels (Figure 7B,C). However, for all cell seeding densities investigated, the amount of TRAIL released from CS only hydrogels was significantly higher (~4–8 fold higher) compared to its release from to CS-CNC hydrogels (Figure 7C). This difference in TRAIL release from the CS-CNC hydrogels could be attributed to presence of electrostatic interactions between the negatively charged CNCs and the positively charged TRAIL protein.

**Figure 8:**
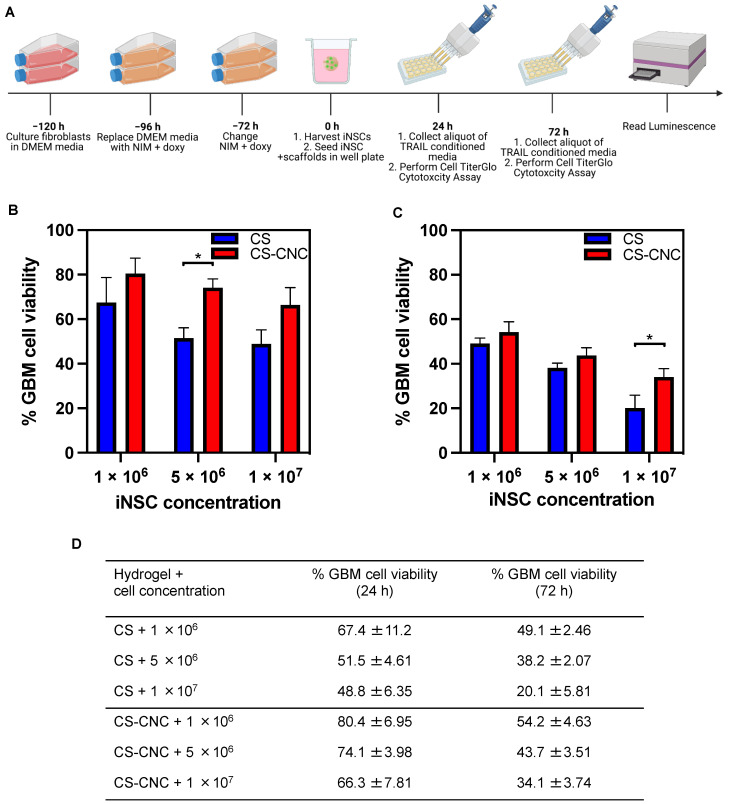
In vitro cell killing activity of NSC-laden CS and CS-CNC hydrogels. (**A**) Schematic illustration of in vitro cytotoxicity experimental design. (**B**) Percent GBM cell viability post 24-h culturing with iNSC-hydrogels relative to non-treated control (*n* = 6, 100 μL samples; * indicates *p* < 0.05). (**C**) Percent GBM cell viability post 72-h culturing with iNSC-hydrogels relative to non-treated control (*n* = 6, 100 μL samples; * indicates *p* < 0.05). (**D**) Summary table %GBM cell viability in NSC-laden hydrogel scaffolds.

Previous studies by Kauer T.M. et al. demonstrated that 50–500 ng/mL of TRAIL was an effective therapeutic concentration to achieve reduction in U87-MG tumor cell viability [16]. Our in vitro TRAIL release studies showed that TRAIL was released at concentrations within the aforementioned range, and as such, we hypothesized that iNSC-laden CS and CS-CNC hydrogel scaffolds should suppress tumor cell growth and proliferation. To investigate this, iNSCs-sTRAIL were cultured for 24- and 72-h and 100 µL of s-TRAIL conditioned media was subsequently collected and seeded against U87-MG tumor cells at cell ratios of 1:1, 5:1, and 10:1 for 24 h, respectively (Figure 8A). As illustrated in Figure 8B–D, results showed that all cell-laden hydrogel scaffolds released TRAIL at concentrations that exhibited efficient tumor cell kill. Moreover, higher kill was observed with all iNSC-laden hydrogel scaffolds (Figure 8B–D).

## 4. Discussion

The most significant challenge with neural stem cell therapy for treatment of post-surgical GBM is the fast clearance observed from the site of implantation. Several commercially available and synthetic-based polymer systems have been investigated as a delivery approach to overcome this clinical limitation [13,14,15,16,17,18]. However, there are several factors to consider when designing a polymer-based scaffold material to encapsulate and deliver neural stem cells. An ideal scaffold material should possess characteristics and properties that maintain maximal retention of stem cells following injection or implantation, enhance persistence overtime, and sustain release of therapeutic stem cells to improve efficacy. Given the aforementioned limitations of existing polymer-based scaffolds for NSCs, we set out to develop an injectable, in situ forming, thermoresponsive hydrogel to improve the encapsulation, retention, and delivery of therapeutic neural stem cells. A biocompatible and biodegradable chitosan-based pH- and thermo-responsive hydrogel system was developed with unique antimicrobial and wound healing properties that may be beneficial at the surgical site. The incorporation of beta-glycerophosphate (BGP) and hydroxyethyl cellulose (HEC) were used as primary and secondary biocompatible gelling agents to promote gelation of the pre-hydrogel mixture under physiological conditions. Furthermore, CNCs were incorporated as a nanomaterial to fine-tune the gelation and degradation kinetics of CS hydrogels. With this technology, we engineered an injectable biomaterial that can be delivered via an intracranial injection post tumor resection to deliver therapeutic iNSCs for treatment post-surgical GBM. This technology was designed as a personalized delivery strategy with the primary goal of incorporating a cancer patient’s genetically derived iNSCs from skin fibroblast for injection into the surgical resection site.

The hydrogel formulation was previously optimized by a series of screening processes to determine the optimal concentration of each component to lead a formulation with osmolarity (300–400 mOsm/kg) and pH (6.5–7.4) that support cell survival (Figure 2) [19]. Our results demonstrate that cell-laden hydrogel scaffolds achieved a gelation of <7 s irrespective of the addition of CNCs and/or cells seeded at a concentration of 1–10 million NSCs/per mL of hydrogel (Figure 3). Although the optimal gelling time of hydrogel scaffolds for intracranial injections is not known, unlike previously reported preclinical studies, these results indicate fast gelling kinetics that can promote maximal retention of neural stem cells following injection. This fast gelling behavior of our hydrogels can be attributed to their composition and crosslinking process in response to temperature and pH changes [19]. Our prior findings have shown that our hydrogels are biocompatible and cytocompatible. This fast-gelling behavior was consistent for all NSC-laden hydrogel formulations at cell densities up to 10 million cells per mL. NSC persistence in the CS-CNC hydrogels have not been determined. Given the importance of NSC persistence on efficacy, future studies will investigate the persistence of NSC-laden hydrogel formulations in vivo. There were notable differences in the degradation profile of cell-free and scaffolds with 1 million cells in comparison 5 and 10 million cells (Figure 5). Cell free and lower cell density scaffolds exhibited a biphasic degradation profile. In the first phase, degradation is governed by diffusion of BGP and loss of water from the matrix and in the second phase, degradation is mediated by polymer-polymer breakdown. It can be noted that higher cell-loaded scaffolds further contributed to enzymatic breakdown of the polymer matrix thus leading to faster degradation of hydrogel scaffolds. The degradation of CS hydrogels was prolonged with the incorporation of CNCs. The slower hydrogel degradation in the presence of CNCs could be correlated to the delayed TRAIL release that was observed with NSC-laden hydrogels containing CNCs. As demonstrated from the SEM images, there were no noticeable differences in porosity of CS only and CS-CNC cell-laden hydrogels. The delayed release of TRAIL could also be attributed to electrostatic interactions of the negatively charged sulfate group on CNCs and the positively charged TRAIL protein. This interaction significantly impacted the in vitro killing activity of CS-CNC iNSC-s-TRAIL-laden hydrogels following 24 h of exposure to U87-MG cells, resulting in GBM cell viability of 66–80%. More importantly, all iNSC-s-TRAIL-laden hydrogel formulations were able to achieve ≥ 50% reduction in tumor cell viability within 72 h. Therefore, our results demonstrate that therapeutic levels of TRAIL are achieved within 72 h following injection.

Unlike other studies that have explored the use of biomaterials for the delivery of NSCs, the present work has focused on elucidating the impact of (1) NSCs on biomaterial properties and (2) biomaterials on NSC functionality [13,14,15,16,17,18]. Although previous studies showed that other scaffold materials possess the ability to enhance in vivo NSC persistence, in the present report we highlight key features and/or properties that can influence overall efficacy of NSC-laden CS and CS-CNC hydrogels. Moreover, our results suggest that these two formulations may behave differently in vivo given the higher burst release of s-TRAIL from CS-only hydrogel compared to a slower s-TRAIL release from the CS-CNC hydrogel. Given the clinical scenario, one hydrogel formulation may be more advantageous for treatment of GBM. In future studies, we aim to test this hypothesis to better understand these differences.

The mechanical properties of the target tissue is an important factor to consider when developing biomaterials for cell and/or drug delivery. The reported storage modulus of the human brain is ~1.81 kPa, and is estimated at ~3.1 kPa and 2.7 kPa for the grey matter and white matter, respectively, [25,26]. Given the relatively low mechanical properties of our CS hydrogels [19], we anticipate a mechanical match to the brain tissue in vivo. Furthermore, the soft mechanical properties in addition to the low immunogenicity and anti-inflammatory properties of CS hydrogels make them ideal systems for brain cancer delivery and regenerative applications [27]. Moreover, the adhesive properties of CS hydrogels may be beneficial to enhance retention of hydrogel in vivo following implantation. In future studies, we will investigate the effects of hydrogels’ mechanical properties on their in vivo safety and efficacy.

In the present studies, the impact of our scaffold on NSC gene expression and overall phenotypic fate has not been explored. It is known that scaffold architecture, stiffness, and chemical properties can influence stem cell fate. Hence, in future studies we aim to investigate the effect of our scaffold properties on iNSC fate and survival. Taken together, this data supports the development of an injectable, in situ forming, thermoresponsive hydrogel to deliver therapeutic neural stem cells for treatment of post-surgical GBM. In future studies, we will also investigate the in vivo safety, persistence, and efficacy of our iNSC-laden hydrogels in post-surgical GBM mouse model. These in vivo studies can help elucidate the clinical benefit of combining injectable biomaterials and therapeutic stem cells for patients eligible for surgery.

## 5. Conclusions

In summary, thermo/pH responsive iNSC-laden CS and CS-CNC based hydrogel scaffolds were successfully engineered and seeded with various cell densities. Irrespective of cell seeding density, CS and CS-CNC hydrogels exhibited instantaneous gelling (<7 s) at 37°C and high viability (>80%) of encapsulated cells. When seeded at the same cell density, CS-CNC hydrogels exhibited prolonged degradation time compared to CS only hydrogels. TRAIL release increased with increasing cell density for both CS and CS-CNC hydrogels and significantly higher release was observed with CS-only hydrogels compared to their CS-CNC counterparts at each cell seeding density. The higher TRAIL release observed with CS-only hydrogels resulted in higher percent tumor cell kill compared to CS-CNC hydrogels. Collectively, this data demonstrates the ability to formulate iNSCs in our chitosan based and chitosan-CNCs hybrid hydrogel scaffolds and maintain cell viability at cell seeding densities up to 10 million cells/mL. To our knowledge, this is the first report on chitosan and chitosan-CNC based injectable and biodegradable scaffolds that can be administered as a stable solution containing clinically relevant human iNSCs and that can promote cell viability and TRAIL secretion and tumor cell kill in vitro.

## 6. Patents

SRB and PM are inventors on a patent application related to this work filed by the University of North Carolina, Office of Technology Commercialization (UNC OTC) (PCT International Application PCT/US2019/034492). SDH is an inventor on patent US11027047B2.

## Data Availability

The data presented in this study are within the article and Supplementary Material, or on request from the corresponding authors.

## Supplementary Materials

The following supporting information can be downloaded at: https://www.mdpi.com/article/10.3390/pharmaceutics14102243/s1, Figure S1: Non-homogeneity of NSCs in hydrogels; Figure S2: In vitro cell viability within hydrogel matrices (*n* = 4); Figure S3: In vitro cell viability within hydrogel matrices (*n* = 3).
