# Supplementary material for: Injectable pH Thermo-Responsive Hydrogel Scaffold for Tumoricidal Neural Stem Cell Therapy for Glioblastoma Multiforme
Source: Pharmaceutics. 2022 Oct 20;14(10):2243. doi: 10.3390/pharmaceutics14102243 (PMC9609352; doi:10.3390/pharmaceutics14102243)
Supplement: Supplementary file 1 [file pharmaceutics-14-02243-s001.zip › pharmaceutics-1954313-SI.pdf]

*pharmaceutics*

*Article*

# Injectable pH Thermo-Responsive Hydrogel Scaffold for Tumoricidal Neural Stem Cell Therapy for Glioblastoma Multiforme

**Jasmine L. King [1] , Panita Maturavongsadit [2], Shawn D. Hingtgen [1] and S. Rahima Benhabbour [1,2],***

[1] Division of Pharmacoengineering and Molecular Pharmaceutics, Eshelman School of Pharmacy, University of North Carolina, Chapel Hill, NC 27599, USA

[2] Joint Department of Biomedical Engineering, University of North Carolina and North Carolina State University, Chapel Hill, NC 27599, USA

* Correspondence: benhabs@email.unc.edu

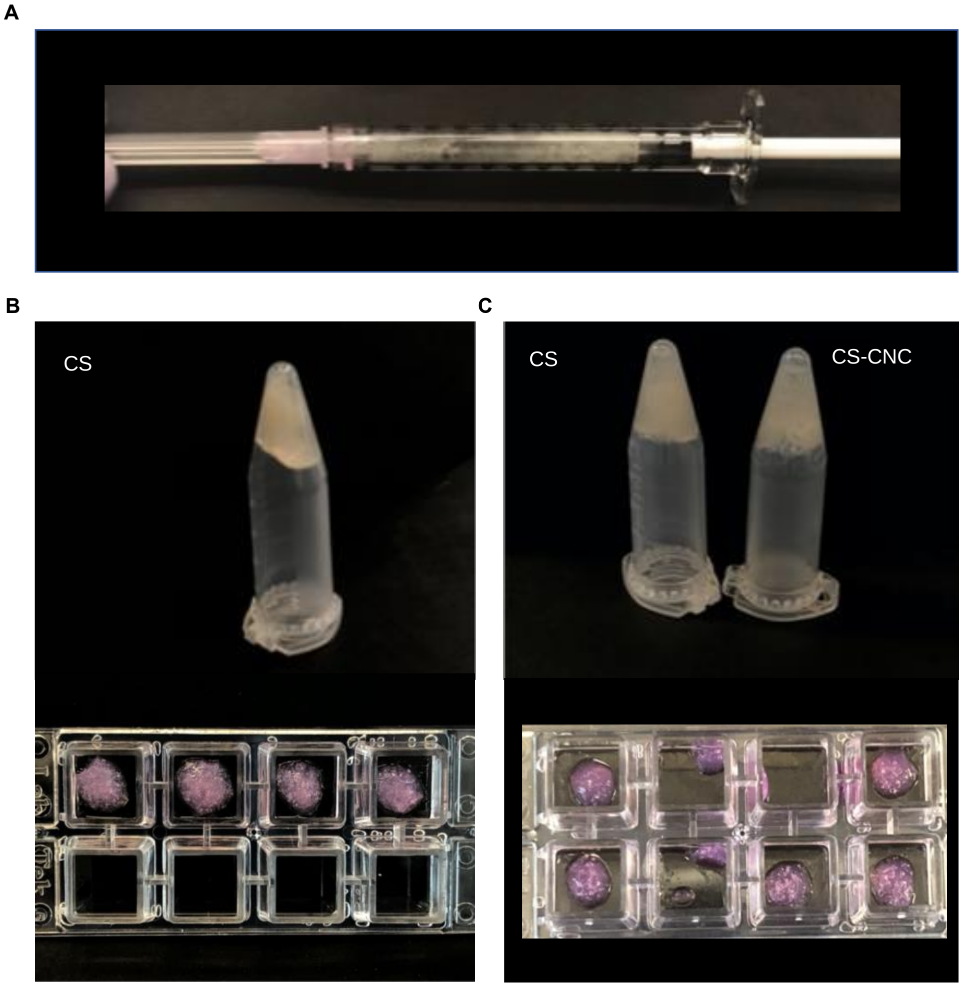


**Figure S1. Non-homogeneity of NSCs in hydrogels.** A) Non-homogenous distribution of 2.5x10$^6$ NSCs in CS pre-hydrogel syringe. B) NSC (2.5x10$^7$/mL hydrogel) clumping within the CS hydrogels post gelation and 24 h following incubation. C) NSC (5x10$^7$/mL hydrogel) clumping within the CS and CS-CNC hydrogels post gelation and 24 h following incubation (n=4; 200 $\mu$L samples).

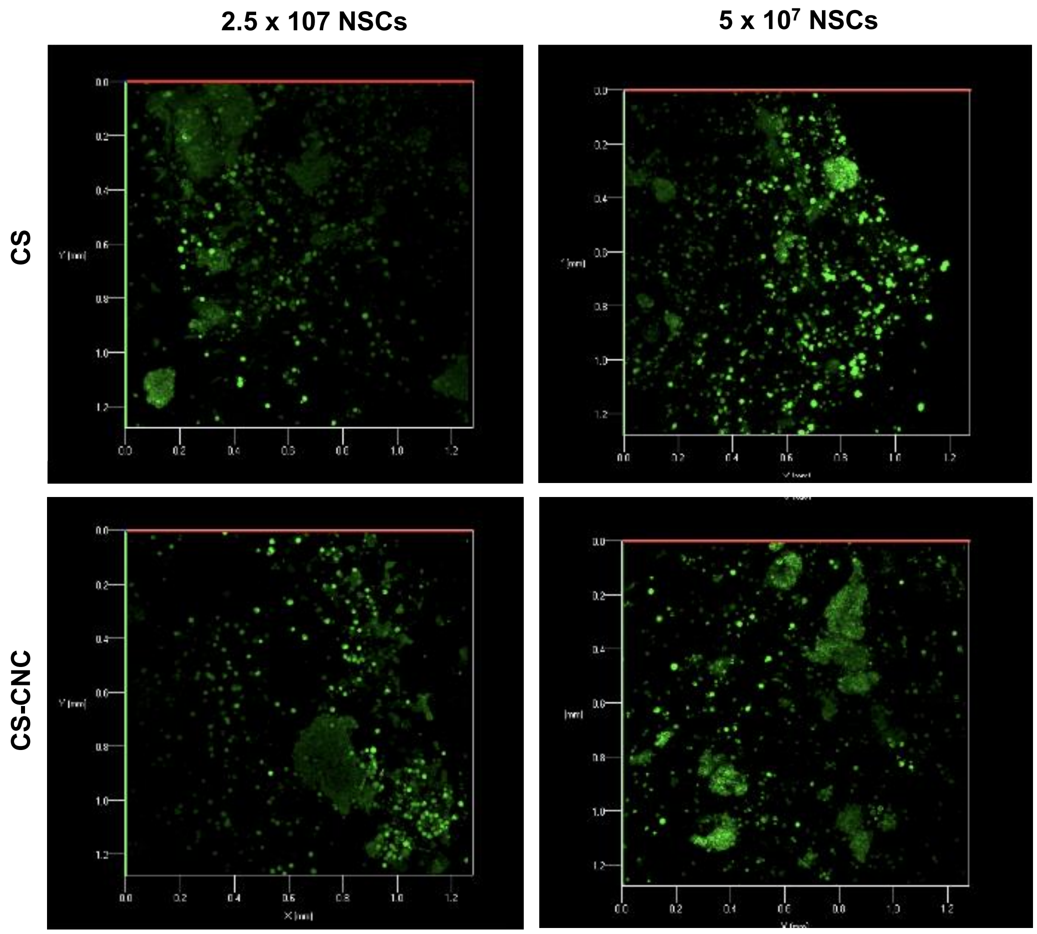


**Figure S2. In vitro cell viability within hydrogel matrices.** Confocal fluorescent images of NSC-hydrogels (2.5x$10^7$ and 5x$10^7$ NSCs/mL hydrogel) showing non-homogeneity and cell-biomaterial clumping post 24 h incubation (n=4; 200 $\mu$L samples).

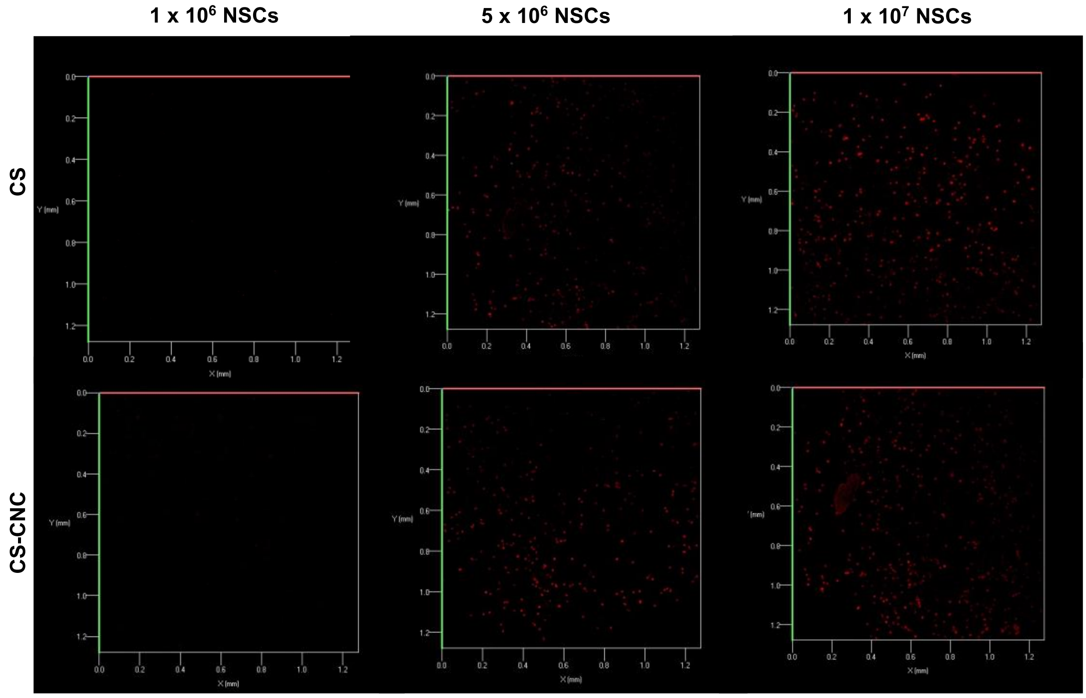


**Figure S3. In vitro cell viability within hydrogel matrices**. Confocal fluorescent images of NSC-hydrogels ($10^6$ - $10^7$ NSCs/mL hydrogel) showing dead cells within the hydrogel matrix post 24 h incubation (n=3; 200 $\mu$L samples).
